# Endoscope-Assisted Cochlear Implantation in Atretic Round Window

**DOI:** 10.7759/cureus.54360

**Published:** 2024-02-17

**Authors:** Muhammad Syafiq H Musa, Khairunnisak Misron, Noor Dina Hashim, Tengku Mohamed Izam Tengku Kamalden

**Affiliations:** 1 Otolaryngology - Head and Neck Surgery, Hospital Canselor Tuanku Mukhriz, Kuala Lumpur, MYS; 2 Otolaryngology - Head and Neck Surgery, Hospital Sultan Ismail, Johor Bahru, MYS; 3 Otorhinolarygology/Otology, Universiti Kebangsaan Malaysia Medical Centre, Kuala Lumpur, MYS

**Keywords:** hearing loss, atresia, round window, endoscopic ear surgery, cochlear implant

## Abstract

Different techniques have been proposed for cochlear implant (CI) from its conventional transmastoid posterior tympanotomy approach. Endoscopy role in the otologic field is still relatively new, but it provides a better surgical view with improved image clarity, especially in the challenging anatomical visualization of the critical structures in CI surgery. A 3-year-old girl with bilateral progressive profound hearing loss was scheduled for left cochlear implant surgery. The pre-operative high-resolution computed tomography (HRCT) of the temporal bone and magnetic resonance (MR) of internal acoustic meatus reported no significant abnormality of the middle and inner ears structures bilaterally. The standard left postauricular cortical mastoidectomy and posterior tympanotomy were performed. However, the microscopic view could not visualize the round window (RW) niche despite a widened extended posterior tympanotomy and surgical field manipulation. Transfacial recess endoscopic examination was done and was able to identify the possibly atretic RW. With endoscopic guidance, CI electrodes were inserted via cochleostomy, and intraoperative impedance measurement and neural response telemetry were obtained both during surgery and the postoperative phase. No intra- and postoperative complications were observed in this case. Following activation, the CI was functioning well. In conclusion, atretic RW is a rare anomaly found intraoperatively during CI surgery. Endoscope-assisted electrode insertion offers excellent visualization of targeted middle ear structures, especially in limited or abnormal anatomy of RW, which could minimize the risk of surgical complications.

## Introduction

Visualization of the round window (RW) during cochlear implant (CI) surgery is essential for correctly positioning the electrodes in the cochlea. The standard transmastoid posterior tympanotomy has become the mainstay approach to the RW since its introduction by House but diversity of the anatomy of the middle ear can be challenging in some cases [1]. The St. Thomas Hospital classification categorizes the accessibility of the RW membrane through microscopic observation into four types: Type I representing 100% visibility, Type IIa for over 50%, Type IIb for less than 50%, and Type III denoting no visual access to the RW membrane [2]. The role of otoendoscopes in middle ear surgery has been extensively explored in recent years. It demonstrates better visibility with its magnified view advantage, which gives the operating surgeon an improved perspective to view hidden areas and in cases of complicated anatomy [3]. Several alternative surgical approaches for CI surgery have been suggested to handle challenging cases effectively. These approaches involve standalone microscopic methods, and more recently, a fusion of endoscopic and microscopic or solely endoscopic techniques has been introduced to assess the RW [4].

## Case presentation

**Figure 1:**
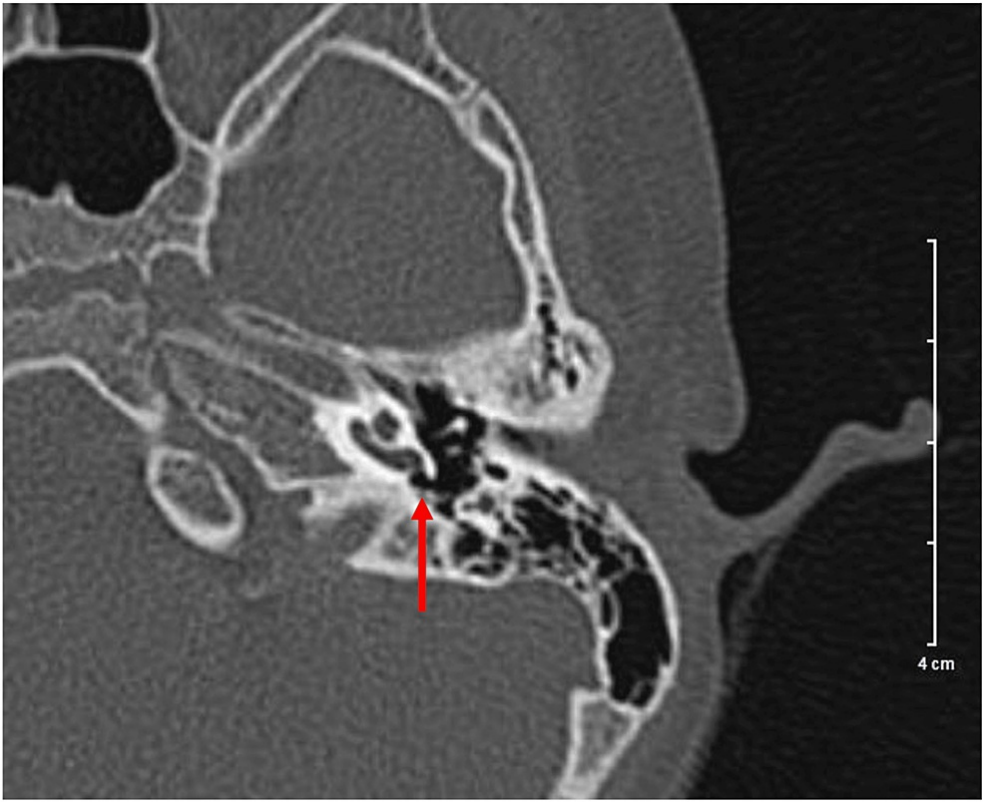
High-resolution computed tomography (HRCT) of temporal bone showed the presence of round window membrane over the left ear as depicted by the red arrow.

A 3-year-old girl was referred for speech delay with suspected hearing loss. Parents noticed that the child could only say limited meaningful words and had poor responses to sounds. Other development progress was up to her age, and there was no significant birth or neonatal history. She has an older sibling who has progressive hearing loss and is currently on a hearing aid. On examination, there was no feature of dysmorphism. Otoscopic examination showed normal external auditory canals and tympanic membranes bilaterally. Other systemic examinations were unremarkable. A pure tone audiogram showed bilateral profound sensorineural hearing loss. Prior to cochlear implantation, high-resolution computed tomography (HRCT) of the temporal bone and magnetic resonance (MR) of the brain and internal auditory meatus were performed and reported as normal findings. The left RW niche was usual in appearance (Figure 1). 

**Figure 2:**
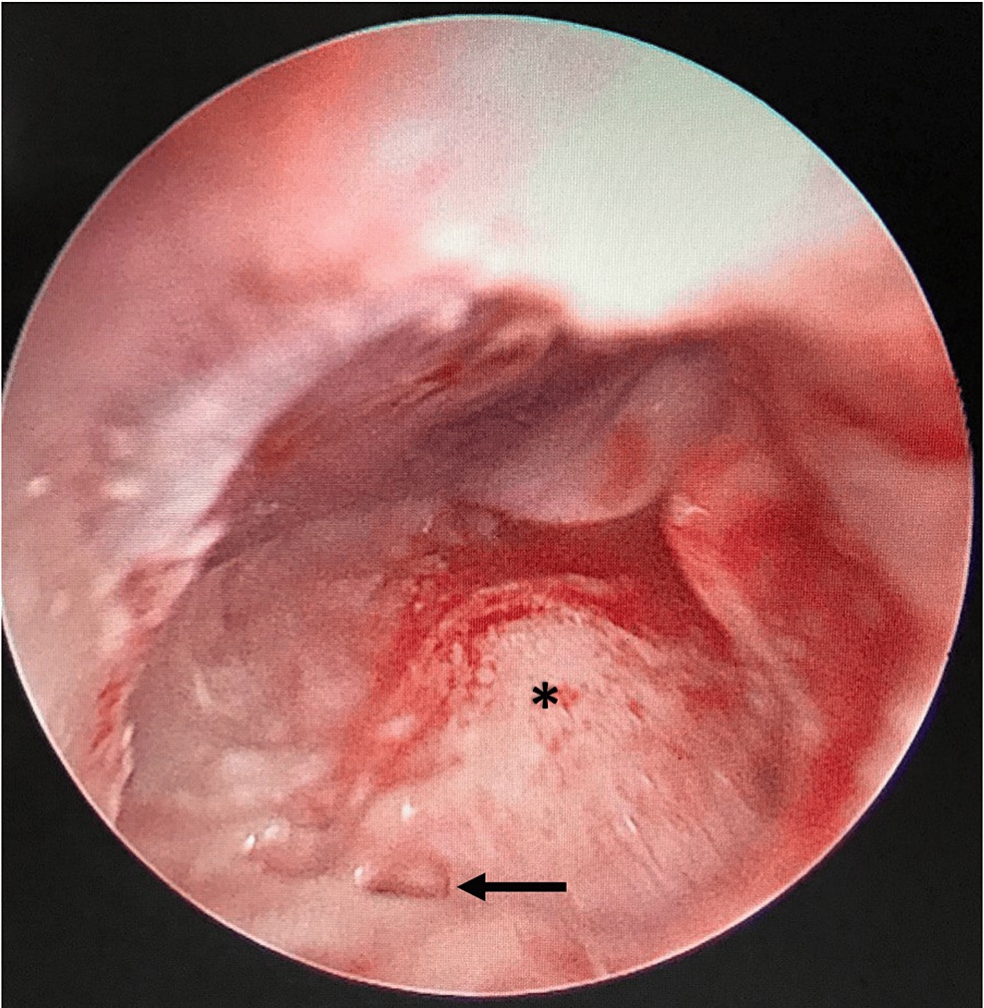
Endoscopic findings of the middle ear via posterior tympanotomy. The black arrow shows the atretic round window plate while the asterisk points to the promontory.

**Figure 3:**
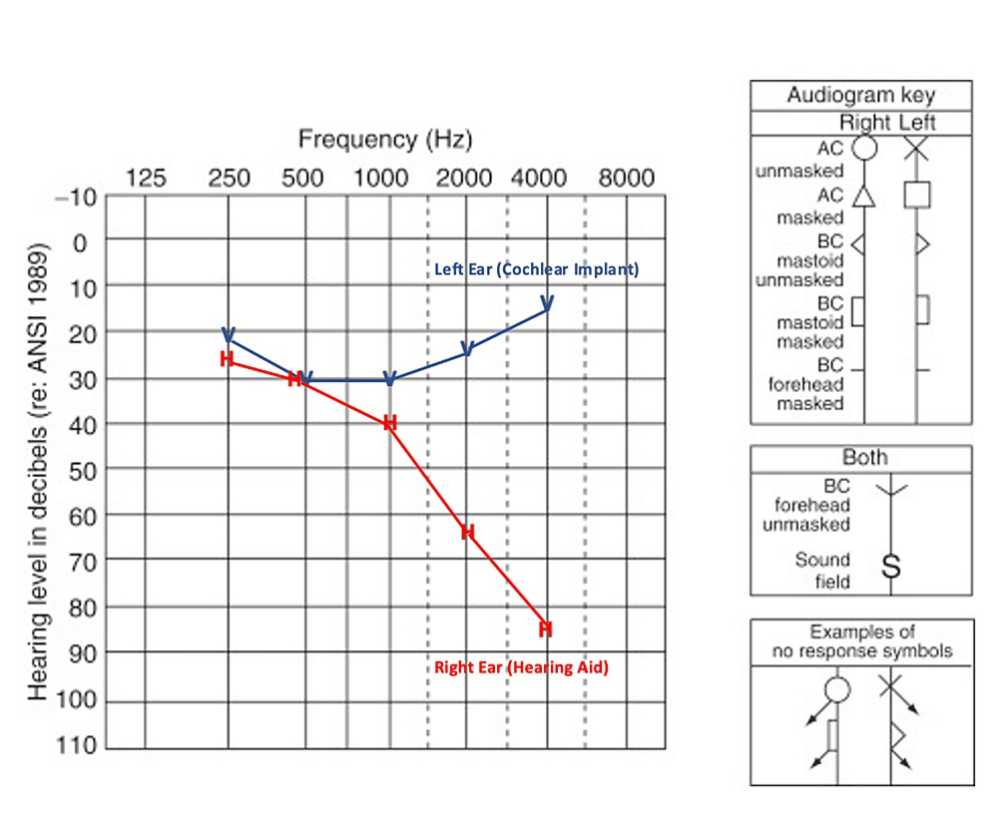
Aided audiogram showing ear-specific hearing thresholds after the surgery (patient was on bimodal hearing). Red H: Right ear-aided thresholds with hearing aid; Blue V: Left ear-aided thresholds with cochlear implant. AC: Air-conduction; BC: Bone-conduction.

She was decided for left CI after a multidisciplinary discussion. She was subjected to the standard postauricular cortical mastoidectomy and posterior tympanotomy using an operating microscope with intraoperative facial nerve monitoring for the implantation. The middle ear structures were identified in their typical locations, specifically promontory, stapedial tendon, and incudostapedial joint. The challenge emerged as the view of the RW niche cannot be well delineated via microscope, and thus, an extended posterior tympanotomy was performed. Despite that, combined with various surgical field manipulations, the RW was still not visualized. A rigid 4 mm 0^o^ endoscope (Karl Storz ®, Germany) was introduced to assist in identifying the RW. Upon gently manipulating the stapes, a reflection of RW was observed, and the RW was identified, potentially seen as atretic RW (Figure 2). Cochleostomy was performed anteroinferior to the suspected bony plate of atretic RW, and 17 out of 20 electrodes (Oticon®, Denmark) were inserted with slight resistance. Intraoperative impedance measurement and neural response telemetry were obtained during surgery and the postoperative phase. There was no intra- or postoperative complication observed in this case. The implant was functioning well upon activation three weeks after surgery (Figure 3).

## Discussion

Cochlear implants have changed the landscape of aural rehabilitation in severe to profound hearing loss patients. The classical technique of the facial recess approach proposed by House in 1967 proved reliable over the years [5]. Round window niche is an important anatomical landmark for CI electrode insertion that provides better placement of the array within the scala tympani while reducing the risk of harming residual hearing [1,6]. Atresia of the RW is rare. Due to its rarity, only a few literature are available regarding this condition, which are mainly case reports. It is usually associated with CHARGE syndrome or other ear abnormalities. On the other hand, non-syndromic RW atresia has been shown to have an association with autosomal dominant inheritance predilection. The clinical feature is mainly conductive hearing loss with an air-bone gap of 30 dB to 40 dB [7]. Interestingly, this patient had bilateral sensorineural hearing loss.

The standard microscope offers multi-planar anatomical visualization but falls short of providing a complete view of the RW, especially when dealing with a tight posterior tympanotomy. The process of CI electrode positioning can prove to be extremely challenging, potentially harming the mastoid segment of the facial nerve, ossicles, and tympanic membrane [5]. Although preoperative radiological evaluation is helpful in determining the direct line of sight of the RW by the microscope, the intraoperative view could be less predictable, especially in children [1,8]. In the demonstrated case, it had been postulated that the RW membrane was likely to be covered by a thin plate of bone, which was not readily seen in the imaging.

Various approaches have been explored in dealing with insufficient microscopical exposure during CI surgery. The role of endoscopy in CI surgery was summarized into endoscopic transmittal or endoscope-assisted approaches. An endoscope-assisted CI approach could be achieved via percutaneous, transcanal, or transfacial recess [8]. Our initial approach using a microscope via posterior tympanotomy could not visualize the RW niche despite extended tympanotomy. The advantage of a magnified view and the ability to look “around the corners’’ using the endoscope has facilitated our transfacial visualization to subsequently discover the atretic RW, a rare middle ear anomaly. This corresponds with the study by Marchioni et al., which advocated the advantage of endoscopy in CI surgery for middle- and inner-ear anomalies [9].

The role of endoscopic-assisted CI has been described in CHARGE syndrome and inadequate RW visualization via posterior tympanotomy [10,11]. However, the technique mentioned in these papers differed from our experience, whereby the transcanal endoscopic cochleostomy was introduced through the tympanomeatal flap to locate the round window. Our technique was quite similar to what had been outlined by Guneri et al., in which the position of RW was identified using an endoscope via extended posterior tympanotomy [8].

The limitation of endoscopic-assisted procedure primarily revolves around the ability of the surgeon's single-handed technique. Besides, the surgeon might have difficulty maneuvering the instruments via posterior tympanotomy with the endoscope in situ. However, these challenges can be overcome with adequate size of the posterior tympanotomy. A recent study by Tarabichi et al. showcased that the posterior tympanotomy approach exhibited the most advantageous trajectory for precise electrode placement; the endoscope-assisted approach could overcome challenging anatomical boundaries faced by the surgeon with minimum intra- and postoperative complications [12]. 

## Conclusions

The auxiliary role of endoscope in a standard posterior tympanotomy approach of cochlear implant surgery has been established previously. Its more expansive use could help surgeons with complicated or abnormal anatomy of the temporal bone such as in this case a rare middle ear anomaly. Combined endoscopic and microscopic surgery is safe with low complications. Adequate size of tympanotomy is essential for visualization of important structures in the middle ear as well as extra space for endoscope introduction during the surgery.
